# Sex-dependent vulnerability for Wistar rats model following intranasal instillation with *Klebsiella pneumoniae* ATCC 43816 causing lobar pneumonia

**DOI:** 10.1186/s41479-024-00126-y

**Published:** 2024-03-25

**Authors:** Patrick Hervé Diboue Betote, Esther Del Florence Ndedi Moni, Sonia Raïssa Gayap Matchuenkam, Sandrine Suzanne Bayengue Beack, Rodrigue Fifen, Raogo Ouedraogo, Gabriel A. Agbor, Rasmané Semde, Nga Nnanga, Maximilienne Ascension Nyegue

**Affiliations:** 1 Laboratory of Drug Development, Centre for Training, Research and Expertise in Drug Sciences, Doctoral School of Sciences and Health, University Joseph Ki-Zerbo, Ouagadougou, Burkina Faso; 2 Laboratory of Pharmacology and Drugs Discovery, Centre for Research On Medicinal Plants and Traditional Medicine, Institute of Medical Research and Medicinal Plants Studies, Yaoundé, Cameroon; 3 Department of Microbiology, Faculty of Science, University of Yaoundé I, Yaoundé, Cameroon; 4 Department of Galenical Pharmacy and Pharmaceutical Law, Faculty of Medicine and Biomedical Sciences, University of Yaoundé I, Yaoundé, Cameroon; 5 Laboratory of Pharmaceutical Technology, Centre for Research On Medicinal Plants and Traditional Medicine, Institute of Medical Research and Medicinal Plants Studies, Yaoundé, Cameroon; 6 Department of Animal Biology and Physiology, Faculty of Science, University of Yaoundé I, Yaoundé, Cameroon; 7 Department of Food Sciences and Technology, Faculty of Agriculture and Veterinary Medicine, University of Buea, Buea, Cameroon

**Keywords:** *Klebsiella pneumoniae*, Intranasal instillation, Lobar pneumonia, Inflammatory granulomas forming, Sex-dependent vulnerability, Wistar rat model

## Abstract

**Background:**

*Klebsiella pneumoniae* has become one of the major threats to public health as it causes nosocomial and community-acquired infections like lobar pneumonia. This infection causes acute inflammation in the lung, characterized by the recruitment of polymorphonuclear cells, generating free radicals, and decreasing the endogenous antioxidant balance system. Many experimental studies have focused on the induction, progression and resolution of infection up to its peak, but these documented processes remain highly random and their sex dependence un-elicited. These fluctuations of physiopathological parameters would impact disease progression depending on the animal’s model and bacterial strain used. The present study investigated the sex-dependent vulnerability of Wistar rats to *K. pneumoniae* ATCC 43816 lobar pneumonia induced by the intranasal instillation method.

**Methods:**

Experimental pneumonia was induced by *K. pneumoniae* ATCC 43816 in male and female Wistar rats following intranasal instillation. The physiopathogenesis of the disease was studied by bacteriological and histopathological exams, histomorphometric analysis of the blood and/or lung tissue, and body weight loss in infected animals. In addition, the overall severity of lesions was determined by the total score obtained by averaging the individual scores from the same group of animals.

**Results:**

The *K. pneumoniae* ATCC 43816 strain showed inoculation dose-, incubation time of the disease- and sex-dependent- differences in its ability to induce lobar pneumonia. Evaluation of different parameters showed that the disease peaked on day 15 post-inoculation, with more pathogenic effects on female rats. This observed sex-dependence difference in Wistar rats was mainly highlighted by the determined lethal dose 50 (LD_50_), bacterial load count in whole blood and lung tissues, body weight loss, inflammatory granulomas forming and diffuse alveolar damages. The pathogenicity was confirmed by scoring the severity of pathologic lesions of lung tissues.

**Conclusions:**

The results obtained highlighted the gender-dependency in the physiopathogenesis processes of *K. pneumoniae* ATCC 43816 induced-lobar pneumonia, in Wistar rats. Female Wistar rats’ susceptibility is useful in studying pathology and in preclinical trial investigations of new treatments for infectious pneumonia.

## Background

Pneumonia is defined as an acute inflammation of the parenchymal structure of the lung [1] and is characterized by increased infiltration of neutrophils, leukocytes, or polymorphonuclear cells, generating free radicals, and decreasing the endogenous antioxidant balance system due to the entrance of infectious agent [2, 3]. On the cell structure of the causative agents, it can be classified as the viral, parasite, fungal or bacterial origin with the Gram-negative preponderance as *Haemophilus influenzae*, *Pseudomonas aeruginosa* and *Klebsiella pneumoniae* in nosocomial pneumonia [4]. Pneumonia caused by *K. pneumoniae* bacteria is characterized by a rapid and progressive clinical course which is often complicated by multi-lobular involvement and lung abscesses [2]. Based on the lungs’ anatomical site of infection, this type of pneumonia can be classified as lobar pneumonia, bronchial pneumonia or acute interstitial pneumonia. Likewise, it can also be classified on the basis of its clinical severity as “no pneumonia”, “pneumonia” or “severe pneumonia” [1, 4].

Worldwide, pneumonia epidemiological data have shown that it provokes death more than any other infectious disease and highlights a slight decrease in prevalence as a result of existing therapies [5, 6]. According to the WHO, data’s from the last two decades on the disease have shown that males have a higher incidence than females [7].

Several studies have investigated sex disparities in the resistance and susceptibility to infection-induced lung injury or the severity of the disease. However, these studies showed a high degree of sex-related variability in pathophysiological processes as reflected in immune and hormonal function in humans and animals [8].

For instance, some studies have shown that males typically exhibit weaker responses compared to females when exposed to infectious pneumonia, exhibiting better resistance to the disease [9]. On the opposite, a more integrative review of the literature on epidemiological data between 2000 – 2020 reveals a higher susceptibility of male subjects to develop infectious pneumonia [6, 7]. Other studies have shown sex-dependent susceptibility and severity of the disease in several animal models of respiratory infection [8–10]. Thus, in animal models of respiratory infection, the severity of the disease could rather be a function of the animal model and microbial strains used in correlation with sex parameters [8].

To date, to the best of our knowledge, very few data are available on the pathogenesis of the induced-respiratory infection in the Wistar rat model. Furthermore, very conflicting data exist regarding the sex-dependent vulnerability of the pathogenesis of *K. pneumoniae*. In addition, many other factors such as the method of induction of the disease, the inducting doses of the pathogenic agent, the incubation time, and the severity of the pathology or the individual resistance to the pathogen, are known to affect the pathogenic profile of *K. Pneumoniae*. Therefore, determining the existing correlations between the sus-mentioned physiopathological conditions could be of great interest in the optimization of preclinical trials on the Wistar rat model for pulmonary infection diseases.

Thus, this study sets out to evaluate the sex-dependent vulnerability of Wistar rats to *K. pneumoniae* ATCC 43816 induced lobar pneumonia by the intranasal instillation.

## Methods

### Bacterial strain

The strain of *K. pneumoniae* ATCC 43816, (obtained by the Head of Bacteriology Laboratories of the University Teaching Hospital of Yaoundé, Cameroon) was used in the study. The strain was identified as *K. pneumoniae* using standard procedures [11].

### Bacterial inoculum

Bacterial strains maintained on the Mueller Hinton Agar slant were grown in static culture in Mueller Hinton broth at 37 ºC for 18 h. Microorganisms were harvested by centrifugation at 2348 g for 15 min, washed three times, and suspended in phosphate-buffered saline (PBS, 0.2 M pH 7.2) to the desired concentration [12].

### Animal housing and acclimation

Wistar rats strain acquired from the Animal House of the Department of Animal Biology and Physiology, University of Yaoundé I, aged 6–8 weeks and weighing 140 ± 10 g at the pneumonia induction onset, were used in this study. The animals were allotted in groups of 6 per cage and housed at 25 ± 3 °C under a 12 h light/dark cycle, except during the designated experimental procedures. They were fed on standard antibiotic-free synthetic feed (Animal Foods, Cameroon). All the experiments were carried out at the Multidisciplinary Laboratory of the University of Yaoundé I. The preclinical experimentation got ethical clearance from the Ethical committee of the University of Yaoundé I ID: 443/UYI/FMSB/VDRC/DAASR/CSD.

### Standardization of the bacterial inoculum for induction of pneumonia

The optimal *K. pneumoniae* dose required for establishing pneumonia in rats was standardised prior to studying the course of pneumonia using McFarland turbidity standard 0.5. To this end, doses ranging from 1.5 × 10^5^ to 1.5 × 10^8^ CFU/mL were administered intranasally. Experimentally, 240 Wistar rats (M/F; 1:1) were used to standardize the pneumonia induction. The grouping of animals is represented in Table 1.

**Table 1:** Repartition of animal for the study

Pneumonia induction time (Days)	Administrated doses in CFU/mL				
	**Control group**	**1.5 × 10**^**5**^	**1.5 × 10**^**6**^	**1.5 × 10**^**7**^	**1.5 × 10**^**8**^
**Day 0**	12	12	12	12	12
**Day 5**	12	12	12	12	12
**Day 10**	12	12	12	12	12
**Day 15**	12	12	12	12	12

Legend: Number of animals in each group: 12** (**M/F; 1/1); **(**CFU/mL): Colony forming unit of *K. pneumoniae* per millilitres of *K. pneumoniae* inoculum solution

Each test group containing female and male Wistar rats was infected with an appropriate dose of *K. pneumoniae* inoculum and animals sacrificed respectively on days 0, 5, 10 and 15 post-inoculations (PI) to count for bacterial load in the blood and lung tissue to determine the infection rate.

The control group used as the not-induction model was also sacrificed on the same days as the tested groups. The inoculum dose that gave the infection rate of 100 per cent, without causing any mortality was taken as the infectious optimal dose [13].

### Induction of pneumonia by intranasal instillation

For intranasal instillation of the bacterial inoculum, the method of Held et al. [13] was employed with slight modifications [2, 14]. Briefly, animals were pre-treated for 3 days by administration of Penicillin (400 000 IU/kg/day) under anaesthesia with Pentobarbital sodium (30 mg/kg intraperitoneal administration) to prevent secondary infections. After a recovery period of 2 weeks, rats were anaesthetized by inhalation of CO_2_ (Medibest, Yaoundé, Cameroon), and administered intranasally with 50 μL of *K. pneumoniae* inoculum or the physiological water while being held in a vertical position. This procedure was performed daily for 15 days. After inoculation, rats regained consciousness and their general state of health and body weight were monitored daily. Each group of rats were sacrificed by disruption of the jugular vein on days 0, 5, 10 and 15 post-inoculations. Whole blood was collected through the jugular vein and immediately diluted 1/10 in aseptic conditions to count *K. pneumoniae* colonies. Lungs were also removed aseptically and divided into two parts. The lungs were cut longitudinally in equal parts. The outer part of the lung was also attributed to the count of *K. pneumoniae* cells. The inner part was dedicated to histomorphopathological analyses.

### Bacteriological examination

1.0 mL of whole blood was prepared by making a 1/10 dilution of the collected blood in sterile PBS (0.2 M, pH 7.2). Concerning the lung tissue, it was sectioned into two halves. The outer part of the lung (1.0 g) was placed in a sterile tube, weighed and crushed. The tissue was homogenized in a Corning glass homogenizer with 9.0 mL of sterile PBS (0.2 M; pH 7.2). Serial dilutions of blood and homogenate lung solutions were plated on MacConkey agar plates (Himedia, India) in triplicate. Plates were incubated at 37 ºC for 24 h, and *K. Pneumoniae* colony counts were determined.

### Histopathological and histomorphometric analysis

The inner part of each lung was fixed for 48 h at room temperature in neutralized 10% buffered formalin, routinely processed and embedded in paraffin [15]. Paraffin-embedded lung sections were haematoxylin and eosin (Sigma Aldrich, France) stained to highlight the structure of lung parenchyma and monitor their inflammation degree [16]. These sections were observed under an Olympus microscope (Olympus U-TV 0.63 × C; SN 9L01588 T2, Tokyo; Japan).

In addition, graduation of the severity of pathological lesions was performed using a section of each animal lung on a semi-quantitative scale of 0 to 4 (Table 2), according to the modified method of Yadav et al. [12]. A total score indicative of the overall severity of lesions was determined by averaging the individual scores from the same group of animals. The Image.J software version 1.53 was used to analyse the histomorphometric data.

**Table 2:** Semi-quantitative scores for grading the severity of pathologic lesions of the lungs

Tissue	Histologic changes	Score
**Lymphatic nodule**	No change	0
	Leukocyte infiltration into inflamed lung tissue (Inflammatory granuloma forming)	+ 1
	Mild inflammatory granuloma without alveolar alterations	+ 2
	Severe inflammatory granuloma with the destruction of alveoli (lung abscess)	+ 3
	Severe inflammatory granuloma with the destruction of alveoli and peribronchial inflammation with luminal slough	+ 4

### Statistical analysis

Results (*n* = 6) are expressed as Mean ± SD. The analysis was done by applying the analysis of variance (ANOVA), followed by Dunnett’s test to compare *K. pneumoniae* colony counts, severity scores of pneumonic lesions in the lung tissues, histomorphometric measurements of inflammatory granulomas areas and body weight evolution on the different days post-inoculation and/or in animal groups. The graphical representation of the data was performed using the Graph Pad Prism 9.3.1 (350) software for Windows (Graph Pad Software Inc., La Jolla California, USA). The difference was taken to be statistically significant at *p* < 0:05.

## Results

### Standardization of lung infection caused by *K. pneumoniae* in rat model

#### Signs of morbidity and lethal dose 50 (LD_50_) of *K. pneumoniae* ATCC 43816 strain

In the first set of experiments, we determined the doses that are able to induce lobar pneumonia by instilling a wide log range of *K. pneumoniae* inoculum (1.5 × 10^5^ to 1.5 × 10^8^ CFU/mL) for 15 days. Thus, the pathogen doses inducing a stable non-mortal infection were determined to lie from 1.5 × 10^6^ to 1.5 × 10^8^ CFU/mL depending on the gender. However, it should be noticed that these doses are the onset of persistent diarrhoea, severe coughing and chills from day 10, post-infection in female rats, whereas in male rats, only the dose of 1.5 × 10^8^ CFU/mL produced the same clinical signs. The results from these experiments allowed us to evaluate the LD_50_ of *K. pneumoniae* ATCC 43816 strain at approximately 1.5 × 10^8^ CFU/mL for female rats, all deaths occurring between day 10 and day 15. Considering that male rats showed any mortality at the studied doses, the LD_50_ can be extrapolated to be higher than 1.5 × 10^8^ CFU/mL.

#### *K. pneumoniae* load in blood and lung tissue

**Fig. 1:**
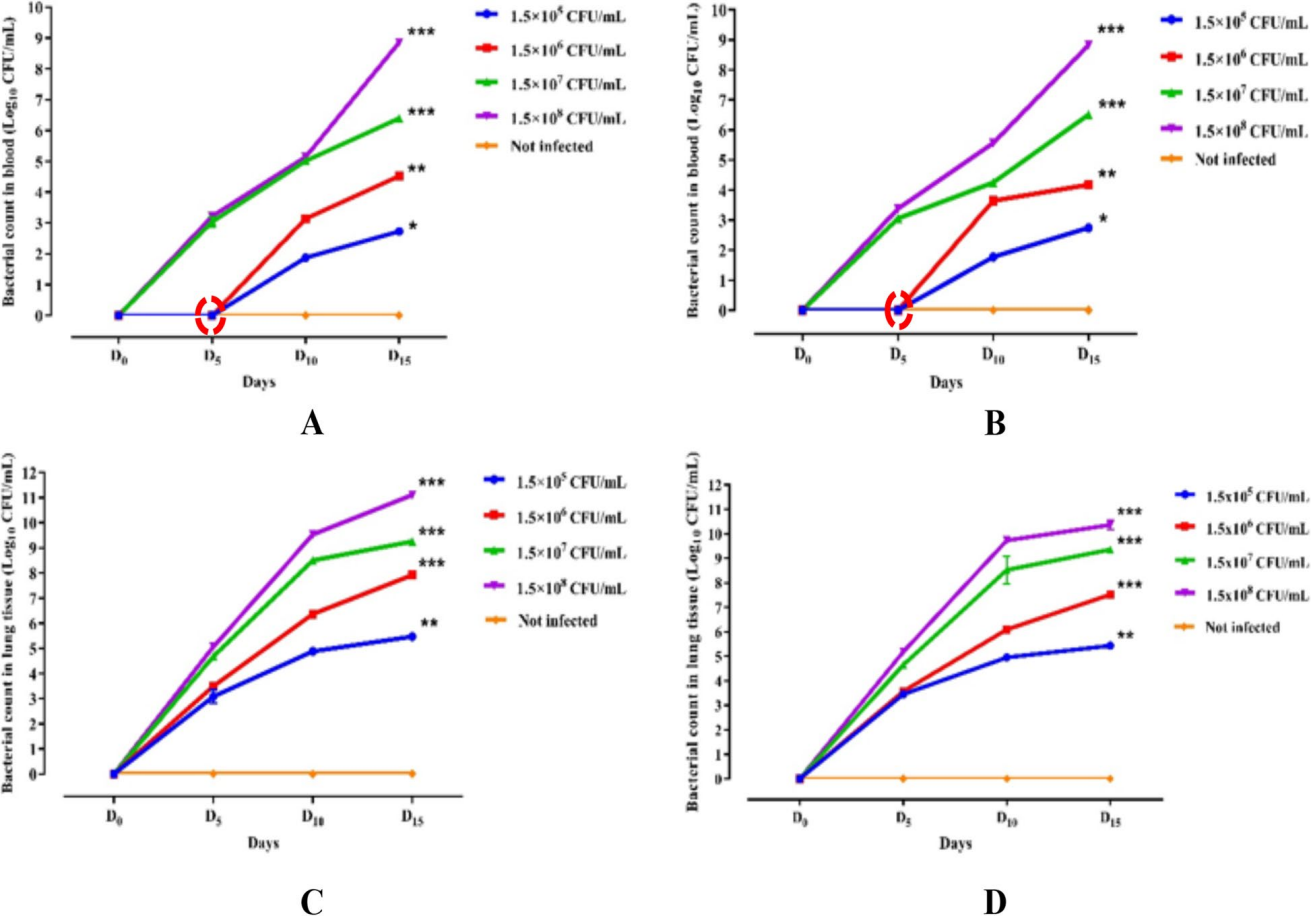
*K. pneumoniae* ATCC 43816 count in the blood and lung tissues of Wistar rats. Bacterial load in the blood (**A**: males and **B**: females) and lungs (**C**: males and **D**: females) of daily up to day 15 instillation of *K. pneumoniae* at 1.5 × 10^5^, 1.5 × 10^6^, 1.5 × 10^7^ and 1.5 × 10^8^ CFU/mL doses in Wistar rats. All results were mean ± SD of six animals per group. Bacterial count evolution was analysed using Dunnett’s post-test (_*_: *p* < 0.05; _**_:* p* < 0.01; _***_:* p* < 0.001); *K. pneumoniae* ATCC 43816 infected groups at 1.5 × 10^5^, 1.5 × 10^6^, 1.5 × 10^7^ and 1.5 × 10^8^ CFU/mL doses vs not-infected group

Bacterial loads in the blood and lungs in female and male Wistar rats were assessed for the sacrifice day (day 0) and the following days 5, 10, and 15 post-inoculations with 1.5 × 10^5^, 1.5 × 10^6^, 1.5 × 10^7^ and 1.5 × 10^8^ CFU/mL of *K. pneumoniae*. The results showed no bacteria detection in blood samples on day 5 after inoculation of 1.5 × 10^5^ and 1.5 × 10^6^ UFC/mL, (*p* > 0.05) both in male and female rats (Fig. 1A–B). However, the infected groups of 1.5 × 10^7^ and 1.5 × 10^8^ UFC/mL of *K. pneumoniae*, indicated bacterial loads of respectively 3.03 log_10_ (± 0.16) and 3.21 log_10_ (± 0.05) bacteria/mL in male rat blood (*p* < 0.05). The bacterial loads for the doses of 1.5 × 10^7^ and 1.5 × 10^8^ UFC/mL were 3.05 log_10_ (± 0.01) and 3.7 log_10_ (± 0.04) bacteria/mL respectively, in female rat blood (*p* < 0.05). On days 10 and 15 PI, *K. pneumoniae* colonies were detected in blood samples at all doses, indicating that these doses induce bacteraemia, after 15 days PI (*p* < 0.05). We noted a proportional evolution between the *K. pneumoniae* load in blood and the tested doses of inoculated bacteria in both male and female rats.

Bacteriological analysis of the lung tissue homogenates showed that *K. pneumoniae* reached the outer part of the lungs on day 5 after inoculation with bacterial concentrations ranging from 3.08 log_10_ (± 0.27) to 5.07 log_10_ (± 0.07) bacteria/mL and 3.45 log_10_ (± 0.03) to 5.19 log_10_ (± 0.05) bacteria/mL for male and female rats respectively (Fig. 1C–D). The bacterial loads regularly increased during the next 15 days PI, reaching 5.43 log_10_ (± 0.04), 8.52 log_10_ (± 0.03), 9.36 log_10_ (± 0.08), and 10.36 log_10_ (± 0.19) bacteria/mL respectively for the doses of 1.5 × 10^5^, 1.5 × 10^6^, 1.5 × 10^7^ and 1.5 × 10^8^ CFU/mL in female rats (*p* < 0.01). A similar evolution of *K. pneumoniae* load in lung tissue was observed in male rats (*p* < 0.01).

### Inflammatory granuloma forming and histomorphometric data

#### Inflammatory granuloma forming

**Fig. 2:**
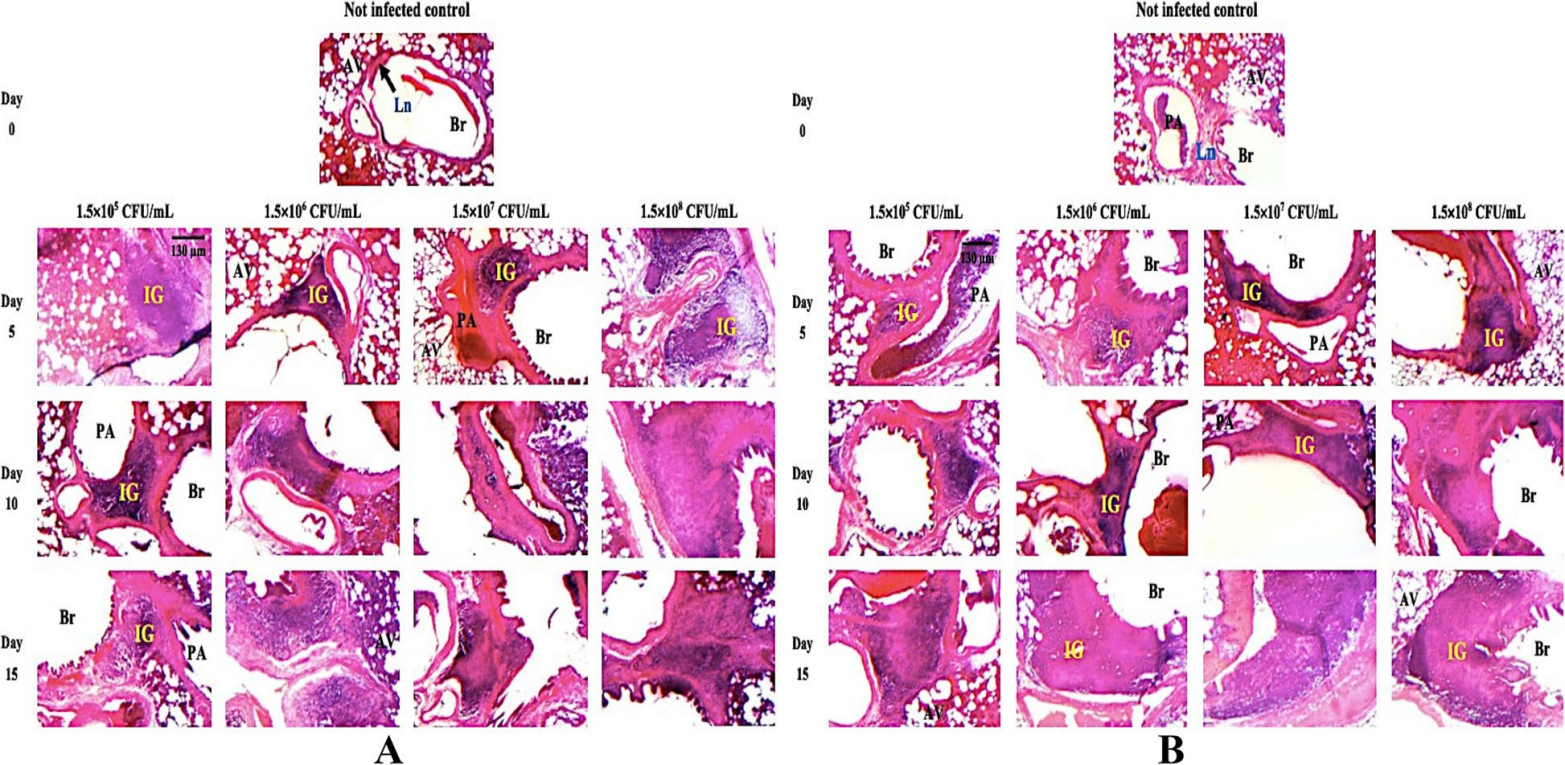
Photomicrographs of lung parenchyma of male (**A**) and female (**B**) Wistar rats. The inflammatory granulomas (IG: in yellow) visualised at days 0, 5, 10 and 15 post-inoculations in the pathologic lungs due to the recruitment of the polymorphonuclear cells by diapedesis towards the inflamed conjunctive tissues of pulmonary parenchyma and showing the abscess formation and destruction of alveoli in the severe stage after *K. pneumoniae* ATCC 43816 infection at 1.5 × 10^5^, 1.5 × 10^6^, 1.5 × 10^7^ and 1.5 × 10^8^ CFU/mL doses. (Haematoxylin & Eosin colouration X40). Br: Bronchiole; Ln: Lymphatic nodules; IG: Inflammatory granulomas; **AV**: Pulmonary alveoli and PA: Pulmonary arteriole

Histopathology of the lungs showed a more diffuse and patchy accumulation of inflammatory cells within the alveolar space along with the infiltrates noted in all lung sections of infected rats (Fig. 2A − B). No exudate was observed in none-infected rats. In addition, the alveoli were found to be intact with undamaged lung parenchyma in not-infected rats.

**Fig. 3:**
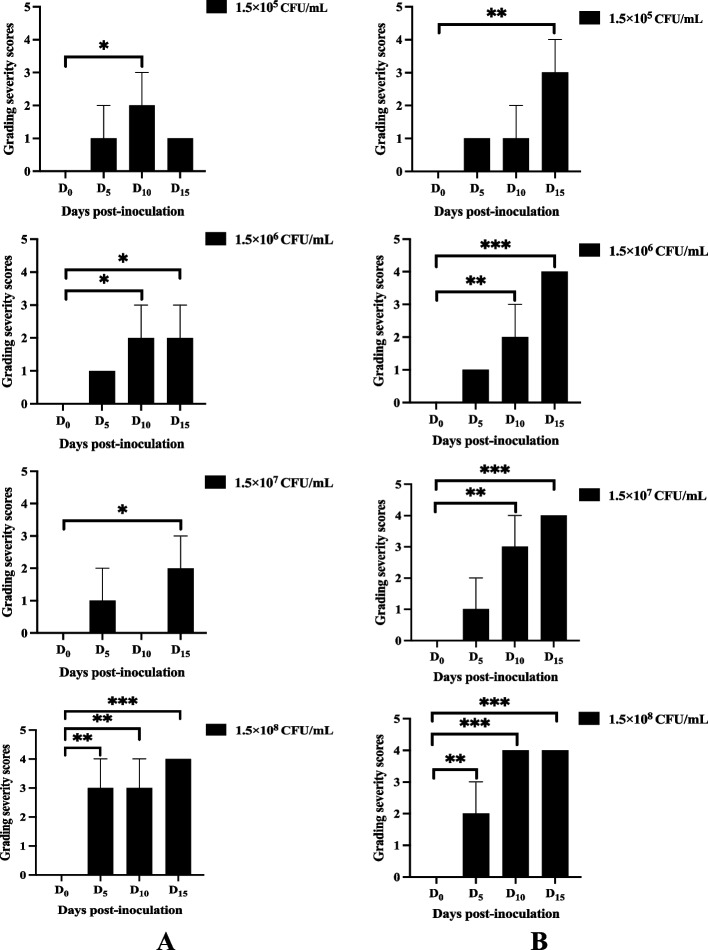
Quantitative scores for grading the severity of pneumonic lesions in lung parenchyma of Wistar rats. Scores of parenchymal lesions of the lungs in male (**A**) and female (**B**) Wistar rats infected by the *K. pneumoniae* ATCC 43816 doses (1.5 × 10^5^, 1.5 × 10^6^, 1.5 × 10^7^ or 1.5 × 10.^8^ CFU/mL) on days 0, 5, 10 and 15 PI. All results were mean ± SD of six animals per group. Severity scores were analysed using Dunnett’s post-test (_*_: *p* < 0.05; _**_:* p* < 0.01; _***_:* p* < 0.001); Days post-inoculation 5, 10 and 15 vs Day 0

Histopathological analysis of the lung tissue represented in Fig. 2B revealed that following 10 days of exposure to *K. pneumoniae* ATCC 43816, female rats developed pneumonia (score 1–4; *p* < 0.001), characterized by cellular infiltrate composed of neutrophils and a few macrophages with abscess formation and destruction of alveoli. The histopathology of female Wistar rats sacrificed on day 15 PI, for all bacterial concentrations, showed installing lobar pneumonia and macrophages dominating (score 3–4; *p* < 0.001) in the affected areas (Fig. 2B). Interestingly, in male rats infected with *K. pneumoniae* ATCC 43816 at 1.5 × 10^5^ CFU/mL, the pneumonia infection was resorbed (score + 1; *p* > 0.05) at day 15, and characterized by the residual presence of leukocytes in the inflamed lung tissue (Fig. 2A). Similar to female rats, only the 1.5 × 10^8^ CFU/mL dose had shown a pattern of pneumonia infection with the intermediate score (score + 3; *p* < 0.01) from day 5 (Fig. 3A–B). The other *K. pneumoniae* ATCC 43816 doses have shown inconsistent infection patterns.

#### Histomorphometric data

**Fig. 4:**
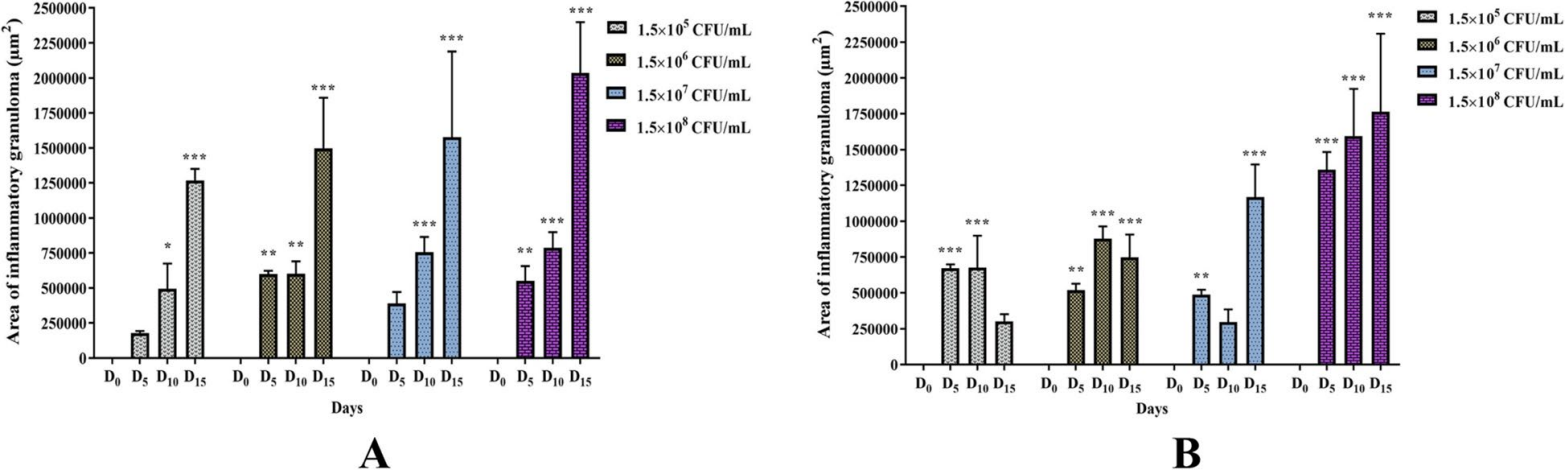
Histomorphometric measurements of inflammatory granulomas areas of lung tissues of Wistar rats. Measures (μm^2^) of parenchymal lesions surfaces of inflamed lungs of male (**A**) and female (**B**) Wistar rats showing the significant difference between each dose (1.5 × 10^5^, 1.5 × 10^6^, 1.5 × 10^7^ or 1.5 × 10.^8^ CFU/mL) on days 0, 5, 10 and 15 PI. All results were mean ± SD of six animals per group. Histomorphometric measurements were analysed using Dunnett’s post-test (_*_: *p* < 0.05; _**_: *p* < 0.01; _***_: *p* < 0.001); Days post-inoculation 5, 10 and 15 vs Day 0

The histomorphometric analysis of inflammatory granuloma areas of lung parenchyma of female Wistar rats also showed that the inflammatory granuloma surfaces are roughly proportional to the *K. pneumoniae* ATCC 43816 inoculated dose, incubation time and post-inoculated bacteria load (Fig. 4B).

Histomorphometric data analysis of inflammatory granulomas surfaces of the male rat lungs at the dose of 1.5 × 10^8^ CFU/mL showed that the spread of infection after days 0, 5, 10 and 15 PI was similar (*p* < 0.001) to that of female rats (Fig. 4A–B). In contrast in the other groups of male rats, the study has shown that the disease evolves in a jagged pattern for doses less than 1.5 × 10^8^ CFU/mL. We noted a significant regression of inflammatory granulomas of 55.44% for infecting dose of 1.5 × 10^5^ CFU/mL (*p* < 0.001) and a slight of 14.95% for 1.5 × 10^6^ CFU/mL dose (*p* > 0.05) at day 15 compared to day 10 PI. In addition, a non-significant decrease of 39.33% was observed from day 5 to day 10 PI with a dose of 1.5 × 10^7^ CFU/mL (*p* > 0.05). It was subsequently; followed by a significant increase in inflammatory granuloma area of 75.69% (*p* < 0.001) observed afterwards from day 10 to day 15 for 1.5 × 10^7^ CFU/mL dose. The proportion of pro-inflammatory cells decreased from day 5 to day 10, at the dose of 1.5 × 10^7^ CFU/mL and from day 10 to day 15 at the 1.5 × 10^5^ and 1.5 × 10^6^ CFU/mL doses, at which doses a decrease, more or less significant of inflammatory granulomas surfaces was recorded, suggesting that bacteria were either cleared from the lungs or disseminated (Fig. 4A).

The increase of inflammatory granulomas area of Wistar male rat lung parenchyma after the inoculation during 15 days of dose of 1.5 × 10^7^ CFU/mL of *K. pneumoniae* ATCC 43816 in this study indicates that bacteria had probably recolonised the parenchyma of lung tissues. For the male rats, the progression of pneumonia following exposure to the same doses wasn’t as homogenous as that observed for the female Wistar rats. However, the transient increase of bacterial loads was associated with moderate macrophage recruitment at bacteria foci on day 5, and the persistence or not of these macrophages up to day 15 post-inoculation highly, would depend on the infecting dose in both sexes.

### Impact of pneumonia infection on the body weight evolution

#### Body weight loss

**Fig. 5:**
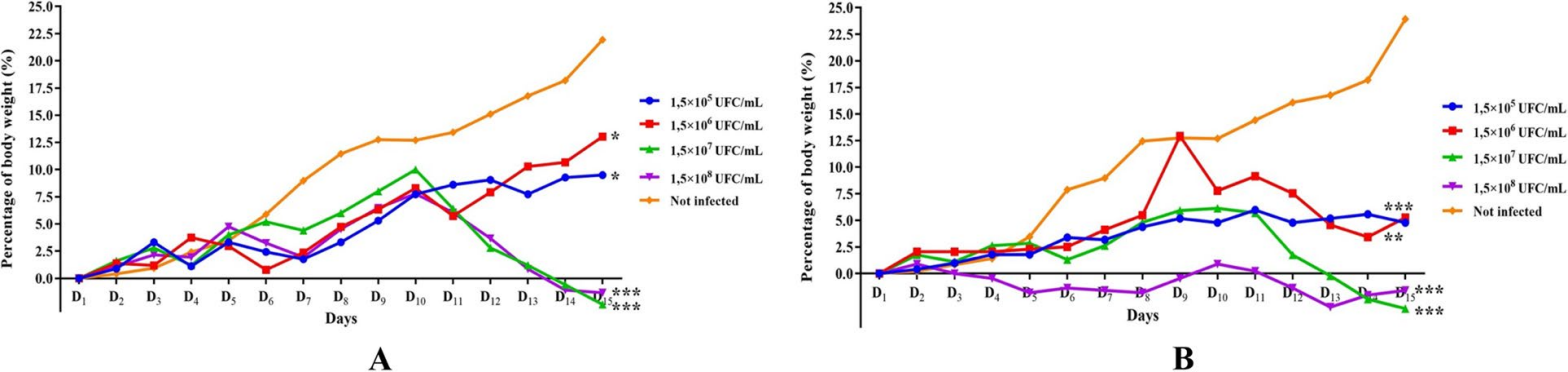
Body weight evolution of Wistar rats. Status of the percentage of body weight of males (**A**) and females (**B**) Wistar rats during *K. pneumoniae* ATCC 43816 pneumonia induction. All results were mean ± SD of six animals per group. Body weight evolution was analysed using Dunnett’s post-test (_*_: *p* < 0.05; _**_:* p* < 0.01; _***_:* p* < 0.001); *K. pneumoniae* ATCC 43816 infected groups at 1.5 × 10^5^, 1.5 × 10^6^, 1.5 × 10^7^ and 1.5 × 10^8^ CFU/mL doses vs not-infected group

The lung infection caused by *K. pneumoniae* ATCC 43816 at 1.5 × 10^5^, 1.5 × 10^6^, 1.5 × 10^7^ and 1.5 × 10^8^ CFU/mL doses provoked a significant body weight loss in female rats at day 9 of inoculation for tested groups 1.5 × 10^6^ CFU/mL (*p* < 0.01) and 1.5 × 10^7^ CFU/mL (*p* < 0.001). Interestingly, the same evolution from day 2 for 1.5 × 10^8^ CFU/mL (*p* < 0.001). In contrast, a slight increase in body weight was observed in female rats infected with 1.5 × 10^5^ CFU/mL (Fig. 5B). However, the body weight increase of this female group remained lower than the none-infected control (*p* < 0.001). For male rats, only the infected groups with 1.5 × 10^7^ and 1.5 × 10^8^ CFU/mL presented a significant decrease (*p* < 0.001) in body weight from day 10, leading to a body weight loss of 12.5% on day 15 (Fig. 5A).

Inoculated *K. pneumoniae* doses that resulted in significant weight loss (*p* < 0.05) in both sexes of Wistar rats were those that also highlighted a regular increase in bacteraemia and/or inflammatory granuloma forming proportional to *K. pneumoniae* ATCC 43816 infective doses.

## Discussion

The pathogenesis of *Klebsiella pneumoniae* in respiratory tract infections has been widely investigated in several studies [12, 13, 17–19]. It is an opportunistic pathogen causing both community-acquired and nosocomial infections [20], ranging from mild urinary tract infections to severe pneumonia with high morbidity and mortality rates [21–23]. Various studies have investigated the role of gender on the infectivity of *K. pneumoniae* or other pneumonic pathogens on animal models of pneumonia, such as mice (Swiss albino, C57BL/6, BALB/c, NMRI, DBA/2, Swiss Webster, etc.) and Sprague Dawley rats [2, 8, 14, 24, 25]. However, previous studies show that these investigations have not been carried out on the Wistar rat model, the experimental model most used for drug discovery studies in tropical areas and, particularly in sub-Saharan Africa due to the ease of this rodent species to acclimating and adapting to environmental conditions.

Especially, the *K. pneumoniae* strains have been used to induce pneumonia in the sus-mentioned animal models with determinate pathogenic characteristics [2, 12, 26]. However, despite the progress in understanding the pathogenesis of this germ, the preliminary research hasn’t focused on understanding the gender impact on the standardisation of the physiopathogenesis of *K. pneumoniae* ATCC 43816 in Wistar rats’ model when used for lobar pneumonia induction. Therefore, this study aimed at the sex-dependent vulnerability of Wistar rat’s respiratory tract infection model using intranasal instillation of *K. pneumoniae* ATCC 43816. Thus, the evaluated parameters in pulmonary tissues and whole blood of *K. pneumoniae* ATCC 43816-infected groups were significantly pathological when compared to the control group, consistent with findings from previous studies [2, 12–14, 27].

Different inoculum doses were evaluated to determine the optimal *K. pneumoniae* ATCC 43816 dose for inducing pneumonia in Wistar rats without causing death. Among the doses tested (1.5 × 10^5^, 1.5 × 10^6^, 1.5 × 10^7^ and 1.5 × 10^8^ CFU/mL), only the animals inoculated with 1.5 × 10^8^ bacteria/mL showed significant lethality in the female group starting from day 12 of inoculation. The optimal dose for inducing stable lobar pneumonia was found to be 1.5 × 10^6^ CFU/mL for female Wistar rats and 1.5 × 10^8^ CFU/mL for male Wistar rats. Animals receiving these doses exhibited a loss of 10% to 12.5% of their initial body weight, indicating the development of lobar pneumonia. In comparison, Vivek-Kumar et al. reported an optimal infectious dose of 1 × 10^6^ CFU/mL *K. pneumoniae* MTCC 109 inducing stable pneumonia in a non-specified male rat model [2]. It is interesting to note that while this result approximately equals the optimal dose that we report in this study for female Wistar rats, it’s a hundred lower than the optimal dose we found for male rats.

Determination of the LD_50_ of *K. pneumoniae* ATCC 43816 inoculum revealed its infective role in pneumonia induction in both male and female Wistar rats, with a lower LD_50_ observed in females. It thus appears that *K. pneumoniae* ATCC 43816 could be more pathogenic and virulent in female rats compared to males. These results align with reports in other previous studies using female C57BL/6 mice inoculated with the pneumonic pathogen *W. chondrophila* [28, 29]. Further similar results were found in studies using various strains of pneumonic pathogens and experimental animal models [2, 9, 29, 30].

It is worth noting that the intranasal instillation technique used in this study has limitations, such as variability in the volume of lung deposition among different animals testing, as some bacteria may remain in the upper respiratory tract, get exhaled, or end up in the digestive tract. Hence, residual bacteria could be either stay in the upper respiratory tract, exhaled, or deviated into the digestive tract [31]. Interestingly, the same evolution of *K. pneumoniae* ATCC 43816 load in lung tissues, as attested by Fig. 1C in the male rats depending on the inoculated doses and inoculation time, was similar to that of the females (Fig. 1D), demonstrating the model’s robustness and the reproducibility of this study’s, and highlighting a low variability of *K. pneumoniae* ATCC 43816 lung deposition. No bacteria were detected in the blood samples of both genders on day 5 post-infection for the lowest doses of 1.5 × 10^5^ and 1.5 × 10^6^ CFU/mL. This could be attributed to bacterial exhalation or the phagocyte action of polymorphonuclear cells like neutrophils and alveolar macrophages recruited to the inflamed lung tissues. In contrast, other studies have reported a significant increase in blood bacterial loads with the same range of doses within 6 h to 1 day post-infection with Gram-negative bacteria, reaching 4.05 log_10_ to 10 log_10_ bacteria/mL in blood samples [2, 12, 25].

In response to this infection, we observed that the *K. pneumoniae* ATCC 43816 proliferation triggers intense inflammation characterized by a disruption of endothelial permeability [30, 32], resulting in purulent fluid release, parenchymal oedema, and a massive influx of polymorphonuclear cells recruited by diapedesis towards the conjunctive inflamed tissues [32]. The gender dependence on *K. pneumoniae* ATCC 43816-induced pulmonary infection is further elicited by histomorphometry images that we used to obtain a spatiotemporal evaluation of inflammatory granulomas and pulmonary oedema caused by the pathogen’s virulence. Indeed, the appearance, amplification and/or regression of the inflammatory manifestations could be observed through the evolution of granuloma areas and histomorphometry of inflammatory granulomas in pulmonary parenchymas during the inoculation period. The quantification of this pathogenic evolution through the graphic representations of granuloma areas (Figs. 2 and 4), indicated the regression of inflammatory granulomas in male rats receiving the inoculum doses of 1.5 × 10^5^ and 1.5 × 10^6^ CFU/mL on days 15, and 1.5 × 10^7^ CFU/mL on days 10 post-inoculation. However, male rats receiving the highest dose of 1.5 × 10^8^ CFU/mL and females across all doses exhibited more regular, stable temporal and spatial evolution in inflammatory granuloma appearance during *K. pneumoniae* ATCC 43816 pneumonia, reflecting an aggravation of the pneumonic infection through the inoculation time. These results could be interpreted as an indication of a greater susceptibility of Wistar female rat vis a vis the pathogenicity of *K. pneumoniae* for the tested infecting doses.

Gender differences in immune function have been well-established in humans and animals [8, 9]. In animal models of respiratory infections, sex has been shown to influence susceptibility and severity of the pneumonia disease, however in inconsistent results [8]. This inconsistency may vary depending on the family or specie of the pneumonic microorganisms used. The results obtained in this study suggest that the examined parameters promoting the pathogenesis of *K. pneumoniae* ATCC 43816 pneumonia in Wistar rats confirm the gender/sex-hormones duality rule. It’s been shown that the impact of sex on *K. pneumoniae* pneumonia pathogenesis always aligns with the influence of sex hormones [9, 10]. Furthermore, males generally exhibit weaker humoral and cell-mediated immune responses compared to females as corroborated by a similar report to this study, showing that female mice tend to be more susceptible to lung infection than males in strains of *Pseudomonas aeruginosa*, a Gram-negative bacteria [33]. Female animals have also been shown to display greater weight loss and significant bacterial load, showing a more vigorous inflammatory response in the lungs than males [8, 33]. However, contrasting results exist showing that male animals could develop more severe inflammatory manifestations than females following pulmonary pathogenic infection. For instance, Carey et al. have shown that male mice develop more severe granulomatous lung lesions than females following infection with Mycobacteria as *Mycobacterium marinum* or *Mycobacteria intracellulare* [8].

These contrasting results give an indication of what could guide the relationship between the animal’s gender and the type and character of specific pro-inflammatory responses. Indeed, based on a general tendency, it has been proposed that males generally exhibit weaker humoral and cells mediated immune responses compared to females [9, 34]. During these pro-inflammatory processes, males would produce an acute alveolar inflammatory response, characterised by predominantly neutrophilic infiltration, oedema and haemorrhage. Females, on the other hand, initiate a chronic peribronchial inflammatory response characterised by mononuclear cell and neutrophil infiltration [10, 34]. It has also been shown that sex hormones and their metabolites (e.g. oestrogen, estradiol, estrone, estriol, progesterone) seem to directly or indirectly influence pro-inflammatory processes and host resistance to the infectious agent [9, 10, 34]. Similarly, Fuseini and Newcomb also showed that oestrogens and their metabolites can trigger lung pro-inflammatory, while male hormones, such as testosterone, usually plays the opposite role [35]. In the case of lower respiratory tract infections, specifically by *K. pneumoniae* pneumonia, these sex hormones induce the overproduction of pro-inflammatory mediators and secretory hyperactivity, which consequently cause aggravation and greater severity of the pathology in female Wistar rats compared to males [9, 33, 34].

Nevertheless, our results show that high infectious doses of *K. pneumoniae* ATCC 43816 (≥ 1.5 × 10^8^ CFU/mL) would over-rule gender and, humoral and cells-mediated influences, over-activating the immune system by the excessive and uncontrolled recruitment of pro-inflammatory mediators in both males and females and leading to the installation of severe pneumonia.

Finally, in relation to the animals’ body weight changes, this study has found no relationship with the animals’ gender. However, a regular increase in bacteraemia, along with a regular increase in the surface of inflammatory granulomas seemed to be accompanied by an inoculum dose-dependent decrease in the animal’s body weight. These results are corroborated by previous studies by Pilloux et al., showing that female C57BL/6 mice infected with *W. chondrophila*, showed a significant body weight loss (*p* < 0.05) along with the appearance and aggravation of the pneumonia disease [14]. Thus, the installation of severe pneumonia induced by *K. pneumoniae* ATCC 43816 seems to be accompanied by a body weight loss, notwithstanding the animals’ gender.

## Conclusion

In summary, the results of this study demonstrate a major difference between the male and the female Wistar rats in the virulence of *K. pneumoniae* ATCC 43816, reinforcing the importance of studying inflammatory granulomas forming, bacteraemia, and diffuse alveolar damages during the physiopathogenesis of lobar pneumonia taking sex and sex hormones into account. Although having highlighted the formation of inflammatory granulomas and alveolar alterations, the study did not differentiate or quantify the inflammatory markers found in infected tissues. These data also show that in the case of lower respiratory tract infections and especially *K. pneumoniae* pneumonia, these sex hormones could promote the overproduction of pro-inflammatory mediators characterized by the appearance of secretory hyperactivity and inflammatory granulomas forming in conjunctive tissue of lung parenchyma, which consequently cause the aggravation of the *K. pneumoniae* pneumonia in female Wistar rats. Considering these results, together with other previous ones, we believe that gender and sex hormones are related to the immune system in a complex manner and influence the pathogenesis of several respiratory tract pathogens, especially *K. pneumoniae*. Furthermore, the relative information to the vulnerability of the Wistar rat model will enable us to better adjust the pathological parameters in future preclinical trials using this animal model in the treatment of infectious pneumonia.

## Data Availability

The datasets used and/or analyzed during the current study are available from the corresponding author on reasonable request.

## Abbreviations

*K. pneumoniae*: *K. pneumoniae* ATCC 43816
LD_50_: Lethal dose 50
Log_10_: Decimal logarithm
M/F: Male/female
PI: Post-inoculation
PBS: Phosphate buffered saline
